# Inhibition of vascular remodelling in a porcine coronary injury model by herbal extract XS0601

**DOI:** 10.1186/1749-8546-1-2

**Published:** 2006-11-23

**Authors:** Hao Xu, Dazhuo Shi, Keji Chen

**Affiliations:** 1 National Integrative Medicine Centre for Cardiovascular Diseases, China-Japan Friendship Hospital, 2 Yinghuayuan East Street, Chaoyang District, Beijing 100029, China; 2 Division of Cardiovascular Diseases, Xiyuan Hospital, China Academy of Chinese Medicial Sciences, 1 Xiyuan Caochang, Haidian District, Beijing 100091, China

## Abstract

**Background:**

Arterial remodelling is a major pathologic change of restenosis after percutaneous coronary intervention (PCI). Our previous studies showed that XS0601 (consisting of Chuangxingol and paeoniflorin) had some effects on the prevention of restenosis after PCI. Therefore, the purpose of this study was to examine whether and how its mechanism was related to the regulation of the arterial remodelling after endothelial injury by balloon dilation.

**Methods:**

Twenty Chinese mini-pigs were randomized into four groups: control, probucol, low-dose XS0601 and high-dose XS0601 group before oversized balloon injury of the left anterior descending coronary arteries. Starting from two days before balloon injury, the mini-pigs in the treated group were administered with probucol (2 g/day) and XS0601 (0.02 g/kg/day for low dose; 0.04 g/kg/day for high dose) for four weeks after balloon injury. The animals receiving balloon injury alone were used as control. Morphometric and angiographic analysis of the injured arteries were performed.

**Results:**

The contribution of intimal hyperplasia and arterial remodelling to angiographic late lumen loss was 41% and 59% respectively. XS0601 markedly inhibited proliferation of smooth muscle cells (SMCs) and transformation of SMCs from contractile to synthetic phenotype in neointima, inhibited hyperplasia-related indices of morphometric analysis and reduce late angiographic lumen loss. The reduction of the late angiographic lumen loss resulting from vascular remodelling was greater after XS0601 treatment.

**Conclusion:**

Both intimal hyperplasia and vascular remodelling are attributed to late lumen loss in this porcine coronary injury model. XS0601 markedly reduced angiographic late lumen loss resulting from intimal hyperplasia, vascular remodelling and XS0601 may be a potential agent to prevent restenosis after PCI.

## Background

Restenosis after percutaneous coronary intervention (PCI), including percutaneous transluminal coronary angioplasty (PTCA) and stenting, is a major factor affecting the long-term success of PCI [1]. Despite numerous trials of pharmacologic interventions, the frequency of restenosis did not diminish [2]. The process of restenosis is not fully understood. Nevertheless, intimal hyperplasia resulting from the proliferation of smooth muscle cells (SMCs) and synthesis of extracellular matrix affect the healing process of an injured artery and restenotic lesion [2-5]. Recently, human and animal studies have shown that vascular remodelling, the change of the total arterial circumference (also called geometric remodelling), is a response to arterial intervention and related to the formation of restenosis [6-8].

The porcine model for studying restenosis has specific advantages over other animal models because of its similarities to the human coronary circulation, particularly the spontaneous development of atherosclerosis and the response to vascular injury [9]. In a balloon injury model of coronary restenosis in normolipemic swine, intimal smooth muscle cell proliferation is histologically similar to the hyperplasia in human restenosis [10]. XS0601 consists of Chuangxingol and paeoniflorin, which are active components of *Ligusticum chuanxiong *Hort and *Paeonia lactiflora *Pall. Our previous study showed that XS0601 restrained the proliferation of cultured SMC stimulated by endothelin (ET) [11]. The purpose of the present study is to observe the effect of XS0601 on vascular remodelling in a porcine coronary injury model and to determine whether it will be a potential agent for preventing restenosis.

## Methods

### Animals and medications

Twenty Chinese mini-pigs weighing between 28 and 36 kg were randomly assigned into four groups. Group 1 (Control) consisted of 5 untreated animals. Group 2 to group 4 each with 5 mini-pigs received different drugs beginning 2 days before balloon injury and continuing throughout the 4-week study period. Group 2 (Probucol) received probucol 2000 mg once a day. Group 3 received 0.02 g/kg of low dose XS0601 (LXS). Group 4 was fed with 0.04 g/kg of high dose XS0601 (HXS). The composition and preparation of XS0601 was the same as previously described [11]. The drugs were crushed to powder and mixed with corn syrup. All animals were fed with normolipemic diet. This study was in compliance with the guidelines of the National Institutes of Health and American Heart Association for the care and use of animals.

### Procedure of balloon injury

All animals were pre-medicated with 325 mg aspirin and 60 mg diltiazem HCl the day before the procedure. The animals were sedated by intramuscular injection of a combination of ketamine (25 mg/kg) and diazepam (1 mg/kg). Half milligram of atropine was given intramuscularly to limit mucous secretions. Anaesthesia was maintained with 2.5% thiopental sodium by administering 2–3 ml every hour. The trachea was intubated. The pigs were allowed to breathe spontaneously unless ventilation became depressed. Adequate anaesthesia was confirmed by the absence of a limb withdrawal reflex or corneal reflex. The ECG and intra-arterial blood pressure were monitored throughout the procedure. Coronary artery injury model was established according to a method slightly modified from Schneider JE, *et al*. [12]. Overstretch-balloon injury was performed with a 20-mm-length PTCA catheter; the inflated balloon-to-artery ratio was about 1.4; the catheter was advanced to mid segment of the left anterior descending coronary artery (LAD). Three 30-second inflations were performed at 10 atm. Between inflations there was one minute deflation period for coronary perfusion. Uninstrumented left circumflex coronary artery (LCx) was used as a control. The animals were sacrificed at the fourth week after the initial overstretch injury, the heart was rapidly excised through a left thoracotomy. The left main coronary artery was perfusion-fixed and serial 4 mm sections were then processed and embedded in paraffin. Cross-sections (4 μm thick) were stained with Verhoeff-van Gieson stains. Angiography was taken at 45 left anterior oblique (LAO) before, immediately after and 4 weeks after PTCA, and 45 right anterior oblique (RAO) views were recorded on video tape for later analysis.

### Morphometric analysis

Each specimen was examined by an experienced cardiovascular pathologist blinded to the treatment group. A lesion grading system based on the criteria [13] listed in Table 1 and computerized color image analysis system (Huizhong Company, China) was applied to morphometric analysis of the narrowest site of the lesion segment. The maximal intimal thickness (MIT, mm) was determined by distance between the internal elastic membrane and the outermost layer of the neointima, circumference of the internal elastic lamina (IELc, mm) and its fracture length (IELf, mm). The extent of injury (injury score, IS) was represented by the IELf, standardized for the size of the artery and expressed by the IELc: IELf/IELc. Area measurements were obtained by tracing the lumen, internal elastic lamina (IEL) and external elastic lamina (EEL): lumen area (LA, mm^2^), area delimited by the IEL (IELa, mm^2^) and area delimited by the EEL (EELa, mm^2^). Thus, media area (MA, mm^2^) obtained by subtracting IELa from EELa, intima area (IA, mm^2^) generated by subtracting lumen area from IELa. The residual lumen (RL) was expressed by lumen area: LA/IELa. Proliferation index (PI) was expressed by IA/EELa. To quantitatively assess the separate contributions of intimal hyperplasia and arterial remodelling to angiographic late lumen loss, according to the methods introduced by Post MJ *et al*. [6], we defined some values as described below. The potential cross-sectional lumen area was calculated from the perimeter of the IEL, assuming a circular configuration. The actual cross-sectional area was obtained by subtracting the area of intima from the potential lumen area. The difference between the radius of potential and actual lumen, derived from their respective cross-sectional areas, was taken as the average thickness of neointima. Arterial remodelling could be obtained by subtracting the doubled average thickness of neointima from late lumen loss. The late lumen loss could be examined by angiographic analysis. The contribution of neointimal thickness or remodelling to late lumen loss could be calculated by dividing late lumen loss by neointimal thickness or remodelling.

**Table 1:** Histopathological grading

**Grade**	**Criteria**
0	No injury noted
1	IEL disrupted; media compressed not lacerated
2	IEL disrupted; media visibly lacerated
3	EEL disrupted

### Transmission electron microscopy

Tissue samples were fixed at 4°C in 2.5% glutaraldehyde, post-fixed in 1% osmium tetroxyde, dehydrated and embedded in Epon 812. The lesion (intima) was located by semithin section. The slice was prepared by ultra-sectioning, stained with uranyl acetate and lead citrate and examined in a JEM-1200EX transmission electron microscope (Japan). The morphologic criteria used for recognition of SMCs were the presence of basal lamina, plasmalemmal vesicles, and myofilament bundles with associated dense bodies. Only those cell profiles of sufficient size to be characterized as SMCs by those criteria were analyzed.

### Immunocytochemical detection of proliferating SMCs

The proliferative profile of the intima was evaluated by immunocytochemical detection of proliferating cell nuclear antigen (PCNA), which is considered to be a valid indicator of cell replication. Paraffin sections from coronary arteries isolated 4 weeks after balloon injury were dewaxed, rehydrated, and boiled for 2 × 5 minutes with antigen retrieval buffer in a microwave oven. Endogenous peroxidase was blocked for 10 minutes with 3% hydrogen peroxide. Sections were incubated overnight with a monoclonal anti-PCNA antibody (ZYMED Company, 1:100 dilution) at 4°C. A biotinylated rabbit anti-mouse secondary antibody was then applied at 37C for 30 minutes, followed by streptavidin peroxidase at 37°C for 30 min. Immunocytochemical detection was visualized with a standard peroxidase enzyme substrate for 3 min. Sections were counterstained with methyl green and mounted with glycerol gelatine. The number of PCNA-positive nuclei and the total number of nuclei within the intima were counted, and proliferation was expressed as the PCNA-labelling index [(number of PCNA-positive nuclei/total number of nuclei) × 100%].

### Angiographic analysis

Angiography analysis was performed by digital calipers on the cinefilms obtained during the baseline study (initial balloon injury) and follow-up study (when the animals were sacrificed). Arterial diameters of the injury site were measured before and after balloon dilations and the balloon-to-artery ratio was calculated. Thus the acute gain and late lumen loss was obtained. The minimum angiographic lumen diameter was measured by an angiographer blinded to treatment groups. The arterial diameters were corrected for angiographic magnification. The magnification factor was determined by dividing the angiographic balloon diameter by the *ex vivo *measured balloon diameter.

### Statistical analysis

Data were expressed as mean ± SD and analyzed by a one-factor ANOVA with STATA 4.0 software. Differences were considered significant at P < 0.05.

## Results

The characteristics of these four groups are listed in Table 2. There was no significant difference in body weight at the time of balloon injury. Rupture of IEL was seen in all animals. The angiographic balloon-to-artery ratios, dilation after injury, the pathologic grades and injury scores based on morphometric analysis were similar among all groups.

**Table 2:** Baseline, angiographic and histopathological characteristics

**Characteristics**	**Control**	**Probucol**	**LXS**	**HXS**
Mini-pig	5	5	5	5
Weight (kg)	32.4 ± 3.36	31 ± 2.74	32 ± 3.32	30.4 ± 2.19
Angiography				
Balloon-to-artery ratio	1.40 ± 0.04	1.37 ± 0.10	1.37 ± 0.09	1.42 ± 0.06
Dilation (after injury) (%)	21.21 ± 0.06	22.24 ± 0.09	24.99 ± 0.09	23.69 ± 0.05
Histopathology				
Pathological grade	2.6 ± 0.89	2.6 ± 0.89	3 ± 0	2.4 ± 0.89
Injury score	0.35 ± 0.18	0.31 ± 0.20	0.31 ± 0.11	0.41 ± 0.20

Values are mean ± SD. No value was significantly different among the four groups by ANOVA (P > 0.05).

### Morphometric analysis

**Figure 1:**
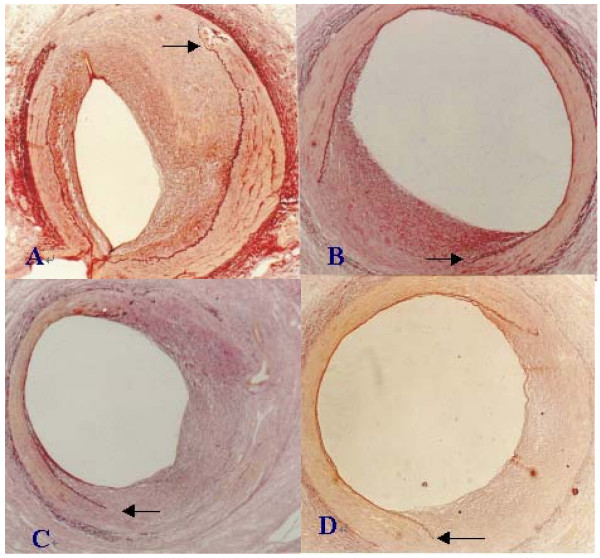
**Representative histological cross sections of coronary arteries 4 weeks after balloon injury (Verhoeff-van Gieson elastin stain, ×50)**. A: Control; B: Probucol; C: LXS; D: HXS. Disruption of the internal elastic lamina (←) was similar at the site of intimal reaction in all groups. Significant reduction of intimal hyperplasia was observed in groups treated with drugs, especially in HXS, compared with control.

There was rupture of the IEL with neointimal growth replacing the disrupted media at 4 weeks after injury (Figure 1). Injury (rupture of IEL) was documented in all animals with similar histopathologic grade among groups (Table 2). General comparison of the characteristics including MIT, injury score, LA, IELa, EELa, MA, IA, RL (%) and PI between treatment and control groups are listed in Tables 2 and 3. Compared with the control group, MIT in HXS is significantly reduced (P < 0.05). As intimal hyperplasia after balloon dilation varied in proportion to injury in this model [13,14], quantitative group comparison was performed and the vessel injury score was used as a covariance. Compared with the control group, in treatment groups HXS or LXS, RL was markedly increased and PI was decreased (P < 0.05 and P < 0.01 respectively), and in HXS, LA was increased four weeks after injury (P < 0.05).

**Table 3:** Morphometric analysis

	**Control**	**Probucol**	**LXS**	**HXS**
MIT (mm)	0.89 ± 0.40	0.54 ± 0.15	0.48 ± 0.08	0.34 ± 0.05*
LA (mm^2^)	0.90 ± 1.18	1.35 ± 0.83	1.88 ± 0.91	2.01 ± 0.88 ▲
IELa (mm^2^)	2.20 ± 0.65	2.16 ± 0.64	2.62 ± 1.03	2.52 ± 0.76
EELa (mm^2^)	3.22 ± 0.63	3.34 ± 0.62	3.68 ± 1.18	3.40 ± 0.64
MA (mm^2^)	1.02 ± 0.11	1.18 ± 0.43	1.06 ± 0.47	0.88 ± 0.30
IA (mm^2^)	1.30 ± 0.68	0.81 ± 0.68	0.74 ± 0.14	0.51 ± 0.27
RL (%)	34.00 ± 37.00	62.00 ± 24.00 ▲	70.00 ± 7.00 ▲▲	77.00 ± 14.00
PI	0.43 ± 0.23	0.24 ± 0.18 ▲	0.21 ± 0.04 ▲▲	0.16 ± 0.09 ▲▲

Values are mean ± SD. *P < 0.05, significance *vs*. control: comparison of group means after ANOVA; ▲ P < 0.05, ▲▲ P < 0.01, significance *vs*. control: comparison of group means after ANOVA taking injury score as covariance.

### Transmission electron microscopy

**Figure 2:**
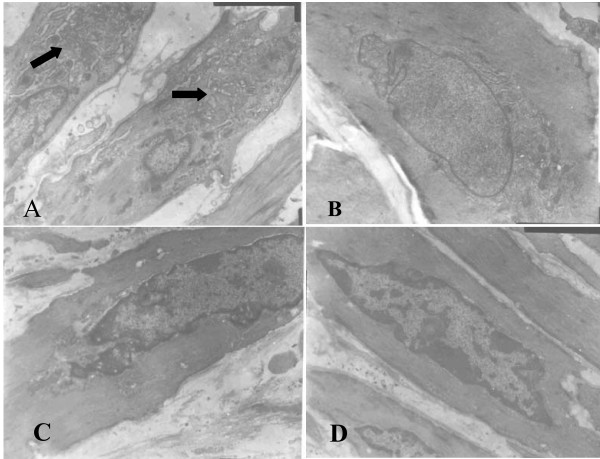
**Representative SMC phenotype in neointima observed by transmission electron microscopy (×8000)**. A: Control; B: Probucol; C: HXS; D: Normal (SMC form tunica media). SMCs of contractile phenotype obtained from tunica media of normal tissue have rich myofilament in cytoplasm and few organelles related to biosynthesis. However, SMCs with synthetic phenotype were more frequently seen in control and they have more organelles (→) related to biosynthesis and fewer myofilament in cytoplasm. Both probucol and XS0601 inhibited transition of SMC phenotype from the contractile to the synthetic, especially in HXS where less increased organelles related to biosynthesis were found.

The IEL was intact in coronary artery of normal porcine. SMCs in tunica media were a predominantly contractile phenotype, manifested by rich myofilament in cytoplasm and only few rough endoplasmic reticulum (RER), mitochondria and free ribosomes around the nucelus. In coronary artery from mini-pigs of control group, IEL was disrupted at location of balloon dilation and SMC transmigrated from media to intima. Hyperplastic SMCs' fibroblasts and few macrophages were frequently seen in neointima. As compared with normal animals, SMCs with synthetic phenotype were more frequently seen in control group, manifested by hypertrophic cell body, increased karyoplasmic ratio, well-developed Golgi's complex, more RER, mitochondria, free ribosomes and fewer myofilament in cytoplasm. Both probucol and XS0601 inhibited the transition of SMC phenotype from contractile to synthetic one, especially in HXS group, which showed less increased organelles related to biosynthesis (Figure 2).

### Immunocytochemical detection of proliferating SMCs

**Figure 3:**
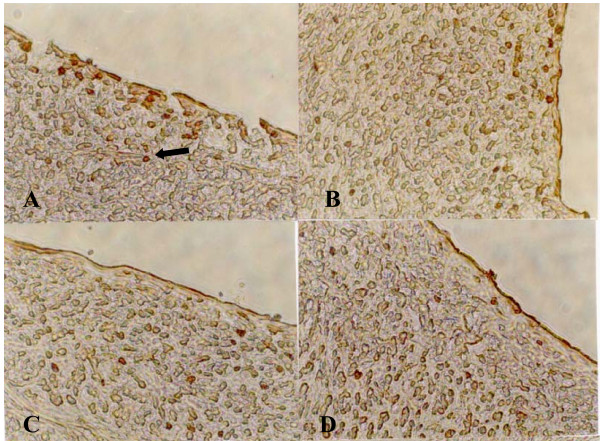
**Immunocytochemical detection of proliferating SMCs in neointima (×400)**. A: Control; B: Probucol; C: LXS; D: HXS. There are more PCNA-positive cells (→) in neointima of injuried coronary artery in control, indicating a state of cell proliferation, than those in different treatment groups, especially in HXS.

**Figure 4:**
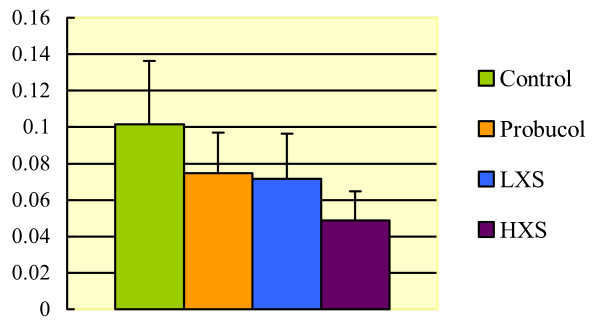
**Comparison of PCNA labelling index in neointima of injury coronary artery among groups**. In HXS, a significant reduction of the proliferative activity in the neointimal layer was indicated by the PCNA labelling index (HXS 4.87% vs. Control 10.14%, P < 0.05).

In HXS, administration of XS was also associated with a reduction of the proliferative activity in the neointimal layer, as indicated by the PCNA labelling index (4.87% vs.10.14%, P < 0.05) (Figures 3 and 4). Although PCNA staining measures the total cell replication activity in the artery segment, injured arteries examined by transmission electron microscopy showed that the proliferating intima was composed primarily of SMCs.

### Angiographic analysis

As presented in Tables 2 and 4, the arterial diameters before and after balloon injury, the balloon-to-artery ratios, dilation and acute gain were similar among groups. All of the drug groups seemed to have decreased late lumen loss and increased the arterial diameters at the fourth week, compared to the control group, but there was no significant difference among them (P > 0.05). Taking acute gain as covariances, both XS0601 and probucol markedly reduced angiographic late lumen loss (P > 0.05) and late lumen loss resulting from intimal hyperplasia (P < 0.01), which was similar to the results obtained with the morphometric analysis. LXS and HXS also significantly increased the arterial diameter at the time of follow-up (P > 0.05) and reduced late lumen loss resulting from remodelling (P < 0.01).

**Table 4:** Angiographic analysis

	**Control**	**Probucol**	**LXS**	**HXS**
Diameter (before, mm)	2.20 ± 0.33	2.05 ± 0.33	2.22 ± 0.49	2.12 ± 0.34
Diameter (after, mm)	2.66 ± 0.34	2.49 ± 0.30	2.75 ± 0.47	2.62 ± 0.45
Diameter (follow-up, mm)	1.34 ± 0.82	1.75 ± 0.47	1.97 ± 0.51 ▲	2.03 ± 0.30 ▲
Acute gain(mm)	0.43 ± 0.17	0.51 ± 0.10	0.58 ± 0.03	0.55 ± 0.11
Late loss (mm)	1.32 ± 0.71	0.74 ± 0.39 ▲▲	0.78 ± 0.33 ▲▲	0.59 ± 0.28 ▲▲

Values are mean ± SD. No values were significantly different between groups by ANOVA (P > 0.05). ▲ P < 0.05, ▲▲ P < 0.01, significance *vs*. control: comparison of group means after ANOVA taking acute gain as covariance.

### Contribution of intimal hyperplasia and remodelling to late loss

As shown in Table 5, the contribution of intimal thickness or remodelling to late lumen loss was not significantly different among groups. From the results of the four groups, 41 ± 20% of the observed angiographic late loss could be explained by intimal thickness, while 59 ± 20% of late loss was due to remodelling in lumen diameter.

**Table 5:** Effect of intimal thickness or remodelling on late lumen loss

	**Control**	**Probucol**	**LXS**	**HXS**	**Combined**
Late loss (mm)	1.32 ± 0.71	0.74 ± 0.39 ▲▲	0.78 ± 0.33 ▲▲	0.59 ± 0.28 ▲▲	
Mean intimal thickness × 2 (mm)	0.71 ± 0.42	0.35 ± 0.29 ▲	0.28 ± 0.03 ▲▲	0.21 ± 0.12 ▲▲	
Discrepancy (remodelling)	0.60 ± 0.39	0.39 ± 0.22	0.50 ± 0.31 ▲	0.37 ± 0.23 ▲▲	
Contribution of thickness (%)	51 ± 15	42 ± 19	44 ± 27	35 ± 19	41 ± 20
Contribution of remodelling (%)	49 ± 15	58 ± 19	56 ± 27	65 ± 19	59 ± 20

Values are mean ± SD. No values were significantly different between groups by ANOVA (P > 0.05). P < 0.05,

## Discussion

The pathogenesis of restenosis is not yet fully understood. However, it is generally accepted that the main initial factors of restenosis are adhesion and aggregation of platelets triggered by vascular injury, leading to the secretion of some chemokines and mitogens such as platelet-derived growth factor (PDGF) and basic fibroblast growth factor (bFGF). These growth factors stimulate the SMC migration and proliferation [15,16], which are considered to be pivotal to the formation of a neointimal lesion. The phenotype transition from the contractile to the synthetic is a morphologic characteristic of actively hyperplastic SMCs. The SMCs with synthetic phenotype react strongly to growth factors, produce and secrete a large amount of extracellular matrix (ECM), and also have capability to migrate and transmigrate [3]. Once the SMCs were activated to change phenotypes, in addition to division growth and ECM secretion, they could produce autocrine or paracrine mediators such as interleukin-1, tumour necrosis factor, PDGF and FGF. Thus, SMCs stimulate themselves and adjacent SMCs to proliferate and form hyperplasia [17]. An investigation showed that the volume index of synthetic organelle of SMCs in lesions from restenosis patients after PTCA was much more than those in *de novo *lesions, which indicated that a great quantity of SMCs were transforming from contractile to synthetic phenotype during the process of restenosis formation [3].

In the present study, obviously thickened neointima was formed after PTCA in this porcine coronary injury model. PCNA-positive cells were highly expressed in neointima and they were reduced significantly in HXS group. This reduction indicated that coronary injury caused proliferation of intimal cells and XS0601 had inhibitory effect on cell multiplication. Results from electron microscopy further demonstrated that SMCs were the most frequently seen cell type in neointima, and that SMCs with synthetic phenotype increased remarkably in neointima in control group. All of the treatment groups could reduce the proportion of SMCs with synthetic phenotype to different extent, especially in HXS group. The results indicated a possible mechanism in preventing restenosis that XS0601 could inhibit the proliferation of SMCs and their phenotype transformation in neointima after coronary balloon injury, and thus reduce intimal hyperplasia.

Recently, vascular remodelling, defined as a structural change in total arterial circumference during the restenosis formation period, is recognized as a major cause of the restenosis [18]. The results from other research groups on different arteries, interventions, species and experimental conditions [6,7] consistently showed that intimal hyperplasia accounts for only a minor proportion of the angiographic late loss in lumen diameter. In our angiographic and histologic study of the Chinese mini-pig fed with normolipemic diet, remodelling accounted for 59% of late lumen loss, and intimal hyperplasia, 41%. Both intimal hyperplasia and vascular remodelling were attributed to late lumen loss in this porcine coronary injury model. Several agents showed potent inhibitory effects on intimal hyperplasia in experimental studies. However, few of them demonstrated their use in preventing restenosis in clinical trials [2]. In our view, arterial remodelling should be considered as a novel therapeutic target, in view of its importance in the development of restenosis. It is accepted that stents reduce restenosis, by improving the initial angiographic result and by withstanding the late pathologic remodelling forces that lead to restenosis after other types of interventions [2]. In addition, all-trans-retinotic acid (derivatives of vitamin A), PP1 (a dual inhibitor of PDGF beta-receptor and Src kinase activity), soluble TGF-betaRII, probucol and endovascular beta-radiation therapy also showed effects on pathologic remodelling in experimental or clinical studies [19-24]. In this study, XS0601 has also shown potential effects on preventing late lumen loss resulting from vascular remodelling.

Since the process of restenosis is involved in multiple pathogenic processes, such as adhesion and aggregation of platelets, thrombosis, migration and proliferation of SMCs, accumulation of collagen, and remodelling. The treatment aiming at only one target may not be enough. The failure of numerous agents for preventing restenosis also suggests the limitation of drugs with only one therapeutic target. Chinese herbal medicines consisting of multiple active components might treat diseases with integrating function on multiple targets. XS0601 consists of the main active factions of *Ligusticum chuanxiong *Hort. and *Paeonia lactiflora *Pall, which are the most commonly used Chinese herbs to improve blood circulation. It has already been demonstrated that the prescriptions for improving blood circulation may regulate blood lipid levels, inhibit the proliferation of SMCs and the activation of platelets, and protect endothelial cells from injury and inhibit lipid peroxidation [25]. Thus XS0601 may prevent restenosis through multiple pathways, which need to be elucidated by further studies.

## Conclusion

The present study found that both intimal hyperplasia and vascular remodelling are attributed to late lumen loss in this porcine coronary injury model. XS0601 markedly reduced angiographic late lumen loss resulting from vascular remodelling. These findings suggest that XS0601 may be useful to prevent restenosis.

## Competing interests

The author(s) declare that they have no competing interests.

## Authors' contributions

HX carried out the immunohistochemistry study, image analysis, performed the statistical analysis, participated in establishment of animal model and drafted the manuscript. DS carried out the angiographic analysis, participated in the design of the study and helped to draft the manuscript. KC conceived the idea of the study, participated in its design and coordination, and gave final approval of this manuscript to be published. All authors read and approved the final manuscript.
